# Development of Advanced Imaging and Molecular Imaging for Barrett’s Neoplasia

**DOI:** 10.3390/diagnostics12102437

**Published:** 2022-10-08

**Authors:** Kaname Uno, Tomoyuki Koike, Waku Hatta, Masahiro Saito, Mizuki Tanabe, Atsushi Masamune

**Affiliations:** Division of Gastroenterology, Tohoku University Hospital, Sendai 981-8574, Japan

**Keywords:** advanced endoscopy, molecular imaging, Barrett’s adenocarcinoma

## Abstract

Barrett esophagus (BE) is a precursor to a life-threatening esophageal adenocarcinoma (EAC). Surveillance endoscopy with random biopsies is recommended for early intervention against EAC, but its adherence in the clinical setting is poor. Dysplastic lesions with flat architecture and patchy distribution in BE are hardly detected by high-resolution endoscopy, and the surveillance protocol entails issues of time and labor and suboptimal interobserver agreement for diagnosing dysplasia. Therefore, the development of advanced imaging technologies is necessary for Barrett’s surveillance. Recently, non-endoscopic or endoscopic technologies, such as cytosponge, endocytoscopy, confocal laser endomicroscopy, autofluorescence imaging, and optical coherence tomography/volumetric laser endomicroscopy, were developed, but most of them are not clinically available due to the limited view field, expense of the equipment, and significant time for the learning curve. Another strategy is focused on the development of molecular biomarkers, which are also not ready to use. However, a combination of advanced imaging techniques together with specific biomarkers is expected to identify morphological abnormalities and biological disorders at an early stage in the surveillance. Here, we review recent developments in advanced imaging and molecular imaging for Barrett’s neoplasia. Further developments in multiple biomarker panels specific for Barrett’s HGD/EAC include wide-field imaging systems for targeting ‘red flags’, a high-resolution imaging system for optical biopsy, and a computer-aided diagnosis system with artificial intelligence, all of which enable a real-time and accurate diagnosis of dysplastic BE in Barrett’s surveillance and provide information for precision medicine.

## 1. Introduction

The incidence of esophageal adenocarcinoma (EAC) has rapidly increased in the western world [1]. Barrett’s esophagus (BE) progresses to EAC with a risk of 0.3% per year, which increases 10–50-fold in dysplastic BE [2,3,4]. The five-year overall survival rate of EAC is about 20%, but early intervention for dysplastic BE with endoscopic ablation and endoscopic mucosal resection (EMR) improves the survival rate up to 90% [5,6]. However, many dysplastic lesions show a flat architecture and patchy distribution in BE, which makes it difficult to detect, even by high-resolution white-light endoscopy (HRWLE) [4,7]. Currently, HRWLE with random 4-quadrant biopsy based on the Seattle protocol (SP) is recommended [8,9], but its adherence in the clinical setting is poor, and the SP has other issues of time, labor, and suboptimal interobserver agreement for diagnosing dysplasia among pathologists [10,11]. As a result, previous studies demonstrated that HRWLE with the SP may miss dysplasia in about 50% of Barrett’s patients with inconspicuous neoplasia [12], suggesting that the current surveillance strategy based on the SP might not be clinically effective for the prevention of Barrett’s cancer [13].Therefore, advanced endoscopic imaging technologies for diagnosing dysplasia in Barrett’s patients are needed. Here, we will review recent developments in endoscopic imaging and envision perspectives for future diagnostic strategies for Barrett’s neoplasia.

## 2. Non-Endoscopic and Endoscopic Technologies

Non-endoscopic or endoscopic technologies, such as cytosponge, endocytoscopy, confocal laser endomicroscopy (CLE), autofluorescence imaging (AFI), and optical coherence tomography (OCT)-related technologies, have been developed, but most of them are not widely used.

### 2.1. Development in Non-Endoscopic Technologies

#### 2.1.1. Biomarker Candidates for Biopsy Specimens

A randomized trial demonstrated that random biopsies based on the SP had low sensitivity for dysplasia in BE patients, because the SP-based biopsy can sample only 4% to 6% of the BE mucosa, leading to missed tiny dysplastic foci [14]. One of the most critical issues was reported to be interobserver variability in diagnosing dysplasia. Additional use of molecular biomarkers for histopathological assessment may improve diagnostic accuracy, so interest in molecular biomarkers has grown, but few biomarkers are ready to use.

First, as for genetic abnormalities, Levine et al. reported that BE and EAC shared common genetic alterations, indicating genetically that BE is a true precursor to EAC [15]. Representative DNA abnormalities in EAC were reported to be aneuploidy, tetraploidy, 17pLOH, and 9pLOH [16].

Next, among protein biomarkers, the p53 protein has the most substantial evidence. Mutations in the p53 gene might induce the overexpression of the p53 protein in the histological assessment, which can heighten interobserver agreement among pathologists [17]. For risk stratification, p53 immunostaining was recommended to be added to the routine histologic assessment of Barrett’s dysplasia [9]. On the other hand, the absence of the p53 pattern in LGD also increases the cancer risk [9,18]. Accordingly, the role of p53 expression in Barrett’s cancer risk is complicated.

Third, previous studies proposed usefulness of other potential biomarkers, including alteration in the expressions of cell-cycle proteins, like cyclinD1 and cyclinA; proliferation markers, like minichromosome maintenance deficient 2, mitochondrial DNA, and telomere shortening; and oncogenes, like HER2/neu, APC, and EGFR [19,20].

Some of these candidates were individually applied for biomarkers in several studies, but multiple biomarkers combined into a single panel were reported to have better sensitivity and specificity than individual biomarkers. Several studies demonstrated that the overexpression of p53, cyclinA, 17pLOH, 9pLOH, and DNA ploidy abnormalities individually might be correlated with dysplasia [17,21,22,23,24], but Galipeau et al. demonstrated that a genomic instability panel, a combination of aneuploidy/tetraploidy, 17pLOH, and 9pLOH, might be a better predictor of EAC than when applied individually [21]. Jin et al. revealed that a gene hypermethylation panel consisting of p16, RUNX3, HPP1, NELL1, TAC1, SST, AKAP12, and CDH13 had high power for cancer prediction [25]. Vithayathil et al. demonstrated that p53 and aneuploidy have a high sensitivity of 81.5% for identifying dysplasia and an area under the receiving operator characteristics curve (AUC) of 0.68 for the prediction of any progression [12]. Accordingly, biomarker panels are expected to improve the diagnostic accuracy or efficiency for BE surveillance with risk stratification. However, these candidates were investigated in a limited number of subjects in a single and retrospective study, so validation studies are needed to translate the biomarker-aided diagnosis from bench to bedside.

#### 2.1.2. Fluorescence in Situ Hybridization for Cytology

Sampling techniques, as well as biomarkers, were developed because dysplastic cells can be scaled more easily than normal epithelium. While the SP can take samples from only about 4–6% of the BE mucosa, pan-esophageal sampling devices, such as brush cytology and Cytosponge (Medtronic, USA), are useful alternatives to detect relevant abnormalities in Barrett’s patients [26]. Although brush cytology can decrease sampling errors from wide Barrett’s mucosa, cytology specimens tend to be more difficult for morphological assessment than conventional endoscopic biopsy [27]. Such inferiority in cytology can be overcome by a fluorescent in situ hybridization (FISH) technique, in which fluoresce-labeled DNA probes specifically identify genetic and chromosomal abnormalities contributing to Barrett’s carcinogenesis. FISH makes it possible to achieve earlier detection of dysplastic BE because genetic alterations might result in morphological changes in the cells. Using brush cytology samples with multicolored FISH probes, Falk et al. showed the amplification/aneusomy of at least one of the two analyzed regions of HER-2 genes in seven of eight cases with Barrett’s cancer, instead of the lack of abnormality in non-dysplastic BE (NDBE) [28]. Additionally, the signals originating from multicolored FISH probes have a strong signal-to-noise ratio, which may facilitate future developments using computer-assisted diagnosis (CAD).

### 2.2. Development in Endoscopic Technologies

New imaging technologies are classified into two systems: (1) wide-field imaging systems to search the entire luminal surface area, and (2) small-field imaging systems with high resolution for optical biopsy.

#### 2.2.1. Wide-Field Imaging Systems

Cross-sectional imaging: Volumetric Laser Endomicroscopy

**Figure 1:**
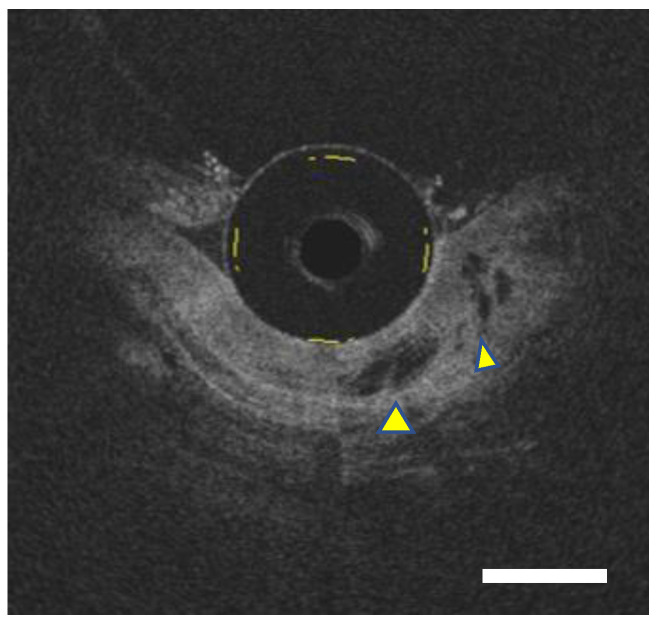
Representative images of probe-type optical coherence tomography (OCT). OCT image (Bar, 1000 μm): OCT images depicted dilated glandular components (yellow arrowhead) underneath the homogenous layer, indicating the existence of the buried Barrett’s glands overlaying the squamous epithelium.

Volumetric Laser Endomicroscopy (VLE) is the second generation of OCT. First, we demonstrate the OCT imaging for BE and Barrett’s neoplasms. OCT forms 2-dimensional cross-sectional images based on differences in the optical scattering of tissue structures, with no need for exogenous contrasts. It provides real-time high-resolution images like low-power microscopy [29]. It depicts surface and subsurface changes, which can be differentiated between normal squamous mucosa, NDBE, and HGD/EAC. We demonstrated buried glands on the probe-based OCT imaging, although HRWLE did not detect them (Figure 1) [30]. The OCT technology is developing as novel cross-sectional imaging for optical biopsy.

As the second generation of OCT, Volumetric Laser Endomicroscopy (VLE) was developed to scan the circumferential area of the esophagus [14]. VLE acquires high-volume data of a large lumen in 96 s and creates 3-dimensional high-resolution imaging of the esophagus wall [31]. VLE provides 6-cm long circumferential components with a depth of 3 mm up to the laminar propria layer. The wide-field imaging by the VLE can increase the detection rate of dysplastic BE. By a one-to-one correspondence between the VLE data and the histology of the EMR specimens, Swager et al. demonstrated that the sensitivity and specificity of the VLE for diagnosing early neoplastic BE was 83% and 71%, respectively [32,33].

In addition, a VLE laser marking system (VLEL) became available for the VLE-guided temporary marking of the mucosa, which is identified for endoscopic biopsy [32]. In a retrospective study, a database with a total of 448 consecutive BE patients was assessed to compare the dysplasia yield among various surveillance strategies, such as (1) SP, (2) VLE without laser marking, and (3) VLEL. The total dysplasia yield was 19.6% in 95 SP, 24.8% in 168 VLE, and 33.7% in 106 VLEL, and both the VLEL group and the VLE groups had significantly higher detection rates of neoplasia (HGD/IMC) than the SP group. Further studies are required to confirm whether VLE can increase the dysplasia diagnosis rate in BE surveillance [34].

In a more recent study using 191 regions of interest (ROIs) from 50 BE patients, Struyvenberg et al. evaluated the diagnostic accuracy of VLE for distinguishing between non-dysplastic and neoplastic ROIs by comparing them with the histological assessment of the VLEL-following biopsies [35]. The accuracy, sensitivity, and specificity of VLE in all ROIs were 79%, 75%, and 81%, respectively. There was no significant association between the VLE experience and the classification accuracy, although the inter-observer agreement was fair (κ = 0.29). The accuracy of the VLE classification was high when investigators diagnosed it with a high level of confidence. However, obscure VLE images, such as the absence of a surface signal intensity or only a subtle increase and partial lack of layering, resulted in incorrect diagnoses of the neoplastic ROIs. Even with high-resolution images, tiny differences between neoplastic and non-neoplastic ROIs in the gray-scale images might be too complicated for human beings to evaluate objectively. Further investigation with artificial intelligence (AI) technology should be conducted to improve the interpretation of the VLE images.

Image-enhanced endoscopy (NBI/AFI imaging)

As for wide-field imaging methods, image-enhanced endoscopy (IEE) imaging with electronic chromoendoscopic techniques can achieve a higher rate of detection and diagnosis than HRWLE.

Narrow band imaging

**Figure 2:**
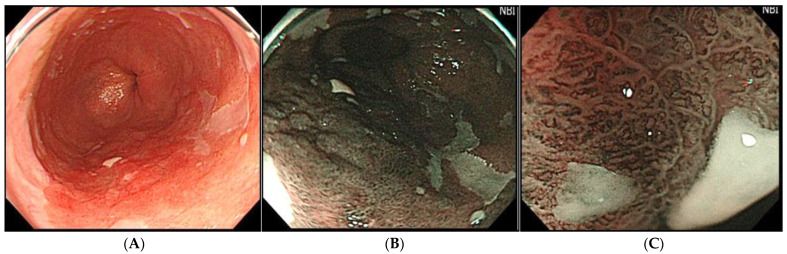
Representative images of superficial Barrett’s adenocarcinoma. (**A**): WLI: The lesion is a reddish nodular protruding lesion in the Barrett’s mucosa of LSBE (Prague classification C4M7), approximately semicircular from the 3 o’clock direction to the 8 o’clock direction. (**B**): NBI: NBI endoscopy showed a brownish-colored elevated lesion. The lesion is brownish compared to the surrounding NDBE, but the demarcation line is indistinct. (**C**): NBI magnifying endoscopy: NBI magnifying endoscopy reveals the irregular capillary vessels, the unstructured mucosa, and the irregularly dilated ductal structure in Barrett’s adenocarcinoma.

Narrow band imaging (NBI) enhances visualization of the glandular and vascular structures in the mucosa [36]. Neoplastic mucosa can be delineated by the irregular structures of the mucosal surface pattern and microvascular pattern, as compared to that of adjacent non-neoplastic mucosa (Figure 2). The microvascular density is gradually increased during the metaplasia-dysplasia-carcinoma sequence of BE [37]. In a nationwide multicenter study in Japan, the sensitivity and specificity of high-definition magnification NBI (M-NBI) for diagnosing dysplastic BE were 87% and 97%, respectively, with high inter-and intra-observer agreements for diagnosing dysplasia of 0.77 and 0.83, respectively [38]. In a Nederland group, the established CAD system for M-NBI images firstly offered an accuracy, sensitivity, and specificity for the detection of neoplastic BE of 84%, 88%, and 78%, respectively, in 30,021 individual video frames, and the following study with a video-based CAD system for 494,364 endoscopic images secondly demonstrated an accuracy, sensitivity, and specificity of 83%, 85%, and 83%, respectively [39]. These studies suggested that M-NBI might be feasible for the diagnosing process by expert endoscopists and deep-learning CAD systems, so M-NBI technology seems to be one of the key technologies for diagnosing Barrett’s neoplasia.

Autofluorescence imaging

Autofluorescence imaging (AFI) provides optical contrast by exciting endogenous substances, such as collagen, nicotinamide, adenine, dinucleotide, and flavin, with short-wavelength light to emit long-wavelength light [40]. AFI distinguishes between BE and Barrett’s neoplasia by the autofluorescence spectrum. Alterations in neoplastic tissue components might cause a loss of the autofluorescence signal, the change of which can be depicted as a purple-red signal (AFI-positive images) within the green background (AFI-negative images). Giacchino et al. demonstrated that the overall accuracy of AFI alone for the detection of HGD/EAC was 57% in BE patients [41]. Boerwinkel et al. showed that AFI possesses a sensitivity of 89% for diagnosing HGD/EAC but with high false-positive rates of 86%, [42] and two cross-over studies also showed that AFI improved the detection of inconspicuous dysplasia but with a high false-positive rate. Unfortunately, Curvers et al. demonstrated that 7 to 21% of BE patients with HGD could not be diagnosed by AFI alone, but by random biopsies, perhaps because of the possible existence of AFI-negative dysplasia [43,44]. Accordingly, such high false-positive/false-negative aspects of AFI alone may not overcome the HRWLE examination with the SP strategy [45]. However, Muldoon et al. revealed that high-resolution endomicroscopy and a nucleus contrast agent, proflavin, could detect neoplastic lesions with 0.87% of sensitivity and 0.85% of specificity [46], suggesting that additional use of other technologies with the AFI examination could be expected to improve its diagnostic power. Next, we will review achievements from the combination of AFI with other technologies, such as NBI, CLE, and molecular imaging.

Combining AFI with other technologies

Trimodal imaging

Endoscopic trimodal imaging (ETMI) was developed by combining AFI with NBI and HRWLE. Additional use of NBI was expected to reduce false positives of the AFI for dysplastic BE [47,48]. However, in a multi-center prospective study, Curvers et al. demonstrated that, although ETMI was more powerful for detecting mucosal abnormalities than HRWLE, the detection rate of neoplastic lesions in the strategy of ETMI with targeted biopsies was inferior to that of HRWLE with random biopsies [43].

Combining AFI with biomarkers

As first demonstrated by di Pietro et al., an AFI-positive signal in BE might be correlated with a field of molecular aberrations independently of dysplasia, indicating that the AFI-positive signals may lack specificity for a malignant potential [45]. Using a large panel of nine molecular biomarkers, including aneuploidy, G2/tetraploidy, 9pLOH, 17pLOH, hypermethylation of p16, RUNX3, HPP1, and IHC for p53 and cyclinA, in combination with AFI in a multicenter study, they then found that AFI-positive areas had an enrichment of gene abnormalities. Furthermore, the three-biomarker panel with aneuploidy, cyclinA, and p53 on the AFI-guided biopsies had a sensitivity and specificity of 95.8% and 88.6%, respectively, for diagnosing HGD/EC, and 74.4% and 94.5%, respectively, for a diagnosis of any grade of dysplasia. The SP had similar sensitivities: 95.8% for a diagnosis of HGD/EC and 84.6% for a diagnosis of any grade of dysplasia. From the point of view that 2.8 biopsies per patient were taken on average compared with 12.8 for the SP, additional use of molecular biomarkers on the AFI-guided biopsies may be useful for Barrett’s surveillance with a significant reduction in the number of biopsies, though perhaps not for improving the diagnosis accuracy of dysplasia [49]. In another retrospective study using ETMI with a five-biomarker panel, i.e., methylation-specific RT-PCR for HPP1, RUNX3, and p16, and IHC for cyclinA and p53, Boerwinkel et al. demonstrated that the expressions of p16, cyclin A and p53 were highly observed in dysplastic BE tissues among the 58 AFI-guided biopsy samples and that the combination of AFI with a biomarkers panel might improve the diagnosis of dysplastic BE [50]. Therefore, a combined assessment of biomarkers on AFI-guided biopsies might be useful for the early diagnosis of neoplastic BE.

On the other hand, some investigators pointed out that inflammation-associated genetic and epigenetic alterations, such as DNA amplification and proliferation in aneuploidy and p53 abnormalities, can cause AFI false positivity, as inflammation can directly and indirectly affect the Barrett’s carcinogenesis [43,51,52]. Therefore, we should carefully interpret abnormal AFI findings from different perspectives.

#### 2.2.2. Small-Field Imaging System

Confocal laser endomicroscopy

**Figure 3:**
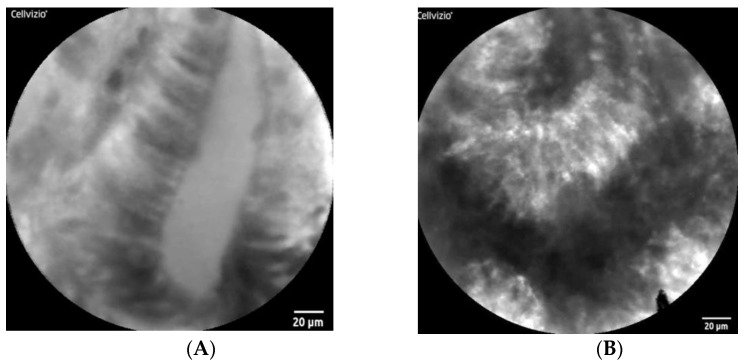
Representative images of pCLE with no administration of tracers. (**A**): Normal epithelium: regular arrangement of epithelial cells with circular nucleus around organized crypt/gland. (**B**): EAC: irregular arrangement of epithelial cells with irregular-shaped nucleus.

Confocal laser endomicroscopy (CLE) enables real-time microscopic analysis of the mucosal microstructure with 1000-fold magnification, despite a narrow view field (Figure 3) [53]. Two CLE systems are currently available, i.e., the probe-based CLE (pCLE) and the endoscopy-integrated CLE (eCLE).

The CLE criteria for diagnosing Barrett’s-associated neoplasia have been established [53,54]. The first study on eCLE for BE patients demonstrated that neoplastic BE was characterized by black cells with irregular borders and shapes, high dark contrast, and irregular leaking capillaries in the mucosa, and that this classification system predicted non-neoplastic and neoplastic BE with a sensitivity of 98.1% and 92.9% and a specificity of 94.1% and 98.4%, respectively, with a high accuracy of 96.8% and 97.4%, respectively, and with excellent inter- and intra-observer agreement [55].

The pCLE criteria were refined in the Miami Classification [56], and several studies demonstrated that the sensitivity and specificity of pCLE for diagnosing dysplasia in BE patients were 75–88.8% and 75–91%, respectively, with good interobserver agreement [57,58]. Barrett’s HGD is characterized by villiform structures, dark irregularly thickened borders, and dilated irregular vessels, while EAC is characterized by disorganized or complete loss of villiform structures and crypts, dark columnar cells, and dilated irregular vessels. In another prospective, double-blind, multicenter study, Wallace et al. mentioned that the sensitivity and specificity of in vivo use of pCLE for diagnosing neoplasia were 88% and 96%, respectively, with substantial agreement, and that there were no differences in the accuracy between experienced observers and non-experienced observers with a structured guidance for the criteria, indicating a short learning curve [58]. As for the diagnostic ability of pCLE for ex vivo EMR specimens that were histologically classified as neoplastic (HGD/IMC) or non-neoplastic (NDBE/LGD), Leggett et al. demonstrated that the sensitivity, specificity, and diagnostic accuracy of ex vivo pCLE for the detection of HGD/IMC were 76%, 79%, and 77%, respectively [59]. These revealed that pCLE has high power for the in vivo diagnosis of neoplastic lesions in BE patients, which were equivalent to those in ex vivo specimens.

However, Gorospe et al. compared the ex vivo diagnostic performance of the pCLE and eCLE systems on fresh EMR specimens in a single center study using the CLEs for the 16 EMRs in 13 patients with Barrett’s HGD/IMC. They demonstrated that the interrater agreements were 0.17 and 0.68 compared to the Miami criteria for the pCLE system and the Mainz criteria for the eCLE system, respectively, and that their accuracy was 37% and 44.3%, respectively [60]. There seemed to be no difference between these modalities in diagnostic abilities, both of which were much lower than in previous studies. They suggested that CLE findings without useful contrast agents might be compromised by large intra- and inter-observer variations. In fact, fluorescein is a non-specific contrast agent for highlighting cellular changes of epithelial cells and subepithelial vasculature up to the lamina propria layer, although it does not stain the nuclei [61]. Therefore, molecular-specific, optically active contrast agents have been developed.

Combining pCLE and other endoscopic modalities

Combining pCLE with wide view field imaging endoscopies may be useful for improving the diagnostic accuracy [62]. Canto et al. showed that the combination of CLE with HRWLE increased the sensitivity for Barrett’s neoplasia from 40% to 96% [63]. As demonstrated by di Pietro et al., a multimodal approach combining pCLE with AFI achieved 96.4% sensitivity and 74.1% specificity for diagnosing BE-related neoplasia, and that additional use of the pCLE on the AFI-targeted areas reduced the false-positive rate of AFI for diagnosing HGD/IMC and any grade of dysplasia from 82.7% and 69.5% to 69.7% and 48.7%, respectively [45].

Moreover, in a multi-center randomized cross-over study of the diagnostic accuracy for inconspicuous dysplastic BE with the AFI-guided pCLE and the conventional HRWLE with random biopsies, Vithayathil et al. described that the AFI-guided pCLE had similar sensitivity and accuracy compared with the conventional protocol, but the biopsy numbers under the AFI-targeted pCLE were much fewer than those of the conventional protocol (mean biopsy numbers: AFI-targeted pCLE vs. SP = 12.3 vs. 2.1) [12]. In this study, the overall sensitivity and specificity for diagnosing any grade of dysplasia of AFI-targeted pCLE were 96.4% and 74.1%, respectively, while AFI-targeted M-NBI had similar specificity (74.1%) with significantly lower sensitivity (57.1%). Additional use of molecular biomarkers (p53, cyclinA, aneuploidy) in mucosal biopsies targeted by the AFI-guided pCLE might improve the accuracy of the diagnosis for inconspicuous dysplastic BE with an AUC of 0.83 compared to those of the conventional protocol. Accordingly, real-time assessment of pCLE on AFI-targeted areas and additional use of biomarker panels might yield a higher diagnostic accuracy for dysplasia with fewer biopsies.

### 2.3. Fluorescence Imaging for Molecular Biomarkers (Molecular Imaging)

Molecular imaging can reveal the biological condition in the tumor microenvironment of Barrett’s neoplasia, together with morphological abnormalities. Alterations in the biological condition might be observed in advance of morphologic changes in the cells. Fluorescence molecular imaging is performed using dyes in the near-infrared range (NIR: wavelength 700–900 nm), which may penetrate deeper into the tissue with less interference by hemoglobin absorption, autofluorescence signals, and tissue scattering, enabling a more precise distinction between a tumor signal and a normal signal [64]. Molecular imaging technologies may revolutionize the Barrett’s surveillance program.

#### 2.3.1. Fluorescence Molecular Endoscopy

Fluorescence molecular endoscopy (FME) targets tumor-specific proteins downstream of oncogenes or tumor suppressive genes by NIR fluorescence [65]. High-resolution FME simultaneously visualizes colorimetric morphological disorders and cancer-specific pathophysiological alterations, similar to in vivo immunohistology [55,66,67,68,69]. A combination of FME with specific biomarkers may achieve an optical biopsy, which is expected to reduce the sampling error and burden in the pathological diagnosis process. However, most FMEs provide microscopic imaging with small view fields. The following development is required to overcome current issues: (1) development of FME: high-quality images sensitive to specific fluorescence signals and without motion artifact of heartbeat and peristalsis; (2) development of tracers: sensitive and specific multifocal gene alterations in heterogeneous characteristics of Barrett’s neoplasia; and (3) development of CAD for “red flags” to alert endoscopists.

#### 2.3.2. Tracers

Molecular imaging enables visualization of morphologic or functional alterations specific to Barrett’s neoplasia to provide individualized information. FME is performed after systemic or topical administration of the tracers. Systemic-administered tracers tend to accumulate more specifically and homogeneously in the targeted area than topical-administered tracers, which are needed to penetrate the mucus barrier to combine with the targets on the cellular surface. However, systemic administration may have the drawback of a longer time from administration to depiction and higher possibilities of side effects compared to topical administration [70]. On the other hand, topically administered tracers, such as lectins or heptapeptides, can reasonably achieve high signal-to-noise and signal-to-background ratios, since most of the proteins targeted for biomarkers translocate from the cytoplasm in normal conditions to the cellular surface in malignant conditions. Topical administration of peptides has an advantage in terms of high affinity with highly biocompatible compounds, low toxicity, and higher tumor penetration. The interval and the manner (local/systemic) of the administration of fluorescent agents may depend on the pharmacokinetic profile and pharmacodynamic properties of the tracers [71,72].

#### 2.3.3. Fluorescence-Labelled Biomarkers Specific for Oncoproteins

There are various attachments for fluorescent dyes, such as nanoparticles (NPs), antibodies, or peptides [73,74]. NPs may be clinically useful because of their properties, such as a spherical shape and small diameter from 10 to 90 nm, and they can distribute widely in Barrett’s mucosa to penetrate biological barriers of the mucosa. Ahmed et al. revealed the diagnostic specificity of FITC-conjugated Muc-2 antibodies for the identification of goblet cells of metaplasia in biopsy specimens from all BE patients [75]. Despite the lack of evidence of the NPs for neoplastic BE, various candidate peptides have been investigated to achieve high sensitivity and specificity for diagnosing Barrett’s neoplasia [65,76,77].

Lectin

As the expression of glycans on the cell surface is increased during Barrett’s carcinogenesis, glycans can be the target for molecular imaging [71]. Glycans can be detected specifically by lectins, which are abundant in a normal diet, heat stable, stable at low pH, and resistant to proteolysis, and their binding may not be affected by fluorescent labeling [78]. With topical administration of wheat germ agglutinin (WGA) as a candidate lectin, Bird-Lieberman et al. demonstrated that the fluorescent signal of lectins decreased during the progression from BE to EAC [71]. Ten minutes after spraying fluorescein-labeled WGA, FME with excitation light between 395–475 nm and emission light between 500–630 nm is depicted in dysplastic lesions of BE, all of which were invisible with conventional HRWLE. Low WGA binding was observed in the HGD lesions in the EMR specimens. The tumor-to-background (T/B) ratio was >10, and the signals from the HGD area were more than 10 times lower than those from the surrounding normal tissue. But inflammation can be a confounding factor and the topically administered probes can be captured in the overlying mucus and mucosal folds, leading to false positives.

ASYNYDA and SNFYMPL

Wang’s group has developed peptide probes, sequences ASY*-fluorescein isothiocyanate (ASYNYDA) and SNFYMPL, with fluorescent labels, which can specially bind to dysplastic Barrett’s mucosa [76,79]. These may allow us to visualize the malignant area in vivo under the FME examination, as well as measure ex vivo the fluorescence intensity of the specimens with fluorescence stereomicroscopy. Previous studies reported the usefulness of the ASYNYDA fluorescence-labeled peptide with in vivo molecular imaging of Barrett’s dysplasia [76,79]. Five minutes after topical administration, pCLE through an accessary channel of an FME depicted the in vivo specific binding to the dysplastic mucosa. Sturm et al. first reported its usefulness in a pilot study of 25 patients [76], and then Joshi et al. applied topical administration to identify the HGD/IMC area with a sensitivity and specificity of 76% and 94%, respectively, in a post-processing method [79].

The peptide sequence SNFYMPL, with homology to the Atrophin-1–like protein, was also investigated in cell lines and EMR specimens [80]. It was bound specifically to malignant OE33 cells and not to non-malignant Q-hTERT cells. On esophageal specimens, the fluorescence intensity of the specimens histologically diagnosed as squamous, intestinal metaplasia, dysplasia, and gastric mucosa was 46.5, 62.3, 100.0, and 42.4 units, respectively. Ideally, topical administration of the ASYNYDA/SNFYMPL peptides can target malignant mucosa on the FME imaging to guide the precise area for sampling or optical biopsy in a large Barrett’s mucosa, but the in vivo evidence is currently insufficient.

Heat-shock protein 70

Fang et al. developed the membrane-bound Hsp70-specific contrast agent tumor-penetrating peptide (Hsp70-TPP). Hsp70 is conjugated with the NIR fluorophore Cy5.5, which offers low autofluorescence, high penetration depth, photostability, and high specificity [77]. The Hsp70-targeted fluorescent signal may accumulate predominantly in malignant lesions, because Hsp70 was reported to be transported from the cytosol to surface membrane through tumor-specific lipid compositions [81,82]. Hsp70-TPP-Cy5.5 was injected intravenously into the L2-IL1B mice 24 h prior to the FME inspection. Flexible FME in L2-IL1B mice in vivo depicted an increase of the Hsp70-derived signal in dysplastic BE/EAC independently of the degree of dysplasia. For the human study, the immunohistochemical study (IHC) demonstrated an overexpression of Hsp70 in dysplastic BE/EAC compared with NDBE, and Hsp70-TPP was also taken up rapidly by human-originated organoids in time-lapse microscopy. Additionally, five minutes after topical administration of the peptide on the EMR specimens, the mean penetration depth of the peptide was about 0.5 mm, and the fluorescence signal intensity in the tumor areas was significantly higher compared with non-tumorigenic mucosa, with a high T/B ratio >4. Therefore, systemic or topical administration of Hsp70-TPP-Cy5.5 can specifically label different stages of dysplastic lesions but not metaplasia or normal tissue. Considering that the membrane expression of Hsp70 is increased in cancer therapy-resistant tumors [82], the Hsp70-TPP expression is expected to improve early detection of EAC as well as monitor the prediction of cancer therapy responses of EAC.

EGFR

The expressions of epidermal growth factor receptor (EGFR), human epidermal growth factor receptor 2 (HER2/Neu, ErbB2), and its downstream molecules (AKT) were observed predominantly in keratinocyte progenitor cells of the esophagus and in 7–34% of primary EAC, indicating that topical administration of the FITC-labeled antibodies on esophageal mucosa can achieve in vivo molecular imaging [73,83,84,85,86]. Realdon et al. reported in vivo detection of EAC after topical administration of labeled antibodies to HER2 [73]. In a rat model for Barrett’s carcinogenesis, the IHC study demonstrated a significant overexpression of HER2 and its downstream AKT in EAC compared to a normal squamous esophagus (9.4-fold) and BE (6.0-fold), and the CLE findings after systemic injection of fluorescent-labeled anti-HER2 antibody in vivo demonstrated heterogeneous overexpression of HER2 in cancer, but with no signal in normal or NDBE tissues.

More recently, Chen et al. demonstrated the usefulness of a multiplexed fluorescence imaging technique by topical administration of two fluoresce-labeled heptapeptides specific for EGFR and ErbB2 [65]. In a pilot study using a peptide QRHKPRE specific for EGFR labeled with Cy5 or KSPNPRF specific for ErbB2 labeled with IRDye800 for 22 BE patients, 92% of neoplastic lesions could be correctly diagnosed with a high T/B ratio with 11% false positives. They demonstrated the feasibility of multiple targets for in vivo early detection of cancers with heterogeneous molecular abnormalities. These studies suggest that fluorescence-labeled antibodies for EGFR/HER2 might be promising targets for molecular imaging of Barrett’s neoplasia.

VEGF

Nagengast WB et al. investigated wide-field near-infrared FME (NIR-FME) using systemic and topical administration of the monoclonal antibody bevacizumab for the vascular endothelial growth factor (VEGFA), labeled with the NIR-fluorescent 800CW [87]. In fourteen patients with Barrett’s dysplasia treated by EMR, their specimens had high expression of VEGFA in all dysplastic lesions. Five patients received an intravenous injection of bevacizumab-800CW two days prior to the NIR-FME, and nine patients received topical administration on the surface immediately prior to the NIR-FME. Topical tracer-based NIR-FME identified four additional dysplastic lesions that were not identified by the HRWLE and NBI findings, and the topical administration led to a slightly higher in vivo T/B ratio > 4 compared with the systemic administration.

Other targets

In this section, we will describe other candidates for molecular imaging.

After the increase in protein expressions of SPARC and SULF1, components of the *Wnt* signaling pathway, were validated among 1976 genes overexpressed in EAC compared to NDBE in the functional genomic mRNA profiling, Zhao et al. developed NIF tracers, SULF1-800CW and SPARC-800CW, which showed higher intensity in Barrett’s dysplasia compared to NDBE by fluorescence imaging on the EMR specimens [88].

Next, Marcazzan et al. demonstrated that the expression of chemokine receptor 4 (CXCR4) on immune cells and epithelial cells was increased during the progression from BE to dysplasia and then to EAC [89]. The CXCR4-targeted cyclic pentapeptide was conjugated to generate the final Sulfo-Cy5-labeled peptide. Four hours after injection with the peptide in six L2-IL1B mice, ex vivo imaging showed that the CXCR4-targeted peptide had accumulated predominantly in the dysplastic lesions with a mean T/B ratio > 2, while a minimal Sulfo-Cy5 signal was observed in normal tissue.

Third, using an FDA-approved PDT photosensitizer to generate a nucleus contrast for molecular imaging, Yeh et al. demonstrated that protoporphyrin IX (PpIX) could strongly distinguish between dysplastic cell lines and metaplastic cell lines with a sensitivity of 95%, specificity of 87%, and an AUC of 0.95 [90]. An irregular nucleus size and the formation of a crowded and non-uniform stratified pattern of the cells were highly consistent with histological dysplasia [91,92]. Considering that PpIX can clinically accumulate at the PDT for HGD patients [90], it may improve the diagnostic ability of molecular imaging for dysplastic BE. However, a PDT photosensitizer was reported to be a useful contrast agent for distinguishing dysplastic cells from metaplastic cells at a single-cell level in a pilot study. Further studies using Barrett’s tissue with heterogeneous cell components will be needed.

2NBDG of a fluorescence marker for cancer metabolism

The administration of 2-[N-(7-nitrobenz-2-oxa-1,3-diaxol-4-yl)amino]-2-deoxyglucose (2-NBDG) was reported to increase fluorescence signaling in cancer cells compared to autofluorescence in normal cells [61]. The uptake of 2-NBDG in cancer cells may be caused by the activation of glucose metabolism through the upregulation of glucose transporters (GLUTs) and its downstream hexokinase enzymes in the cytoplasm of glandular cells during carcinogenesis, although 2-NBDG is not taken up in goblet cells of metaplastic mucosa [93,94,95,96,97]. An irregular structure and high intensity in the gland in the 2NBDG-enhanced CLE findings might be associated with morphologic and metabolic features in neoplastic mucosal cells. Additionally, 2-NBDG has a low molecular weight of 330kDa, which may penetrate the sub-surface cell layers by topical administration [98,99]. Based on the image criteria of neoplastic BE (HGD/EAC) or metaplastic BE (metaplasia-LGD), 2NBDG-enhanced imaging achieved a sensitivity of 96% and specificity of 90% for diagnosing neoplastic BE and AUC of 0.96–0.97, as topical 2-NBDG administration might provide quantifiable images to discriminate neoplastic sites from metaplastic sites with high sensitivity and specificity [61]. In another study, Gorospe et al. also demonstrated that 2NBDG-fluorescence intensity was closely correlated to the degree of dysplasia with diagnostic accuracy and interrater agreements of 78.6% and 0.87%, respectively [60]. Accordingly, FME imaging with topical 2-NBDG administration may provide sufficient quantifiable data to develop a real-time CAD system in the future.

However, the 2-NBDG has two issues for application in a clinical setting. First, a longer incubation time is necessary for acquiring the fluorescence images. Second, infiltration of inflammatory cells in the lamina propria may be falsely positive because 2-NBDG is not a specific molecule for Barrett’s carcinogenesis. If these drawbacks are overcome, molecular imaging with 2-NBDG may be advantageous in wide-field imaging for red flags like 18-fluorodeoxyglucose.

### 2.4. Development of Computer-Aided Detection and Computer-Aided Diagnosis

Recent improvements of CAD technologies have dramatically improved endoscopic diagnosis. Given a large dataset, a computer algorithm for deep learning may be able to develop criteria mathematically optimized for diagnosing Barrett’s neoplasm [100]. Previous studies demonstrated that the CAD system for diagnosing early Barrett’s neoplasms in the HRWLE images achieved a sensitivity and specificity of 0.86% and 0.87%, respectively [100,101]. In this section, we will reveal recent CAD developments in advanced imaging techniques, such as molecular imaging and VLE.

#### 2.4.1. CAD for OCT/VLE

Tiny differences between neoplastic and non-neoplastic ROI in the gray-scale images might be too complicated for human beings to objectively evaluate high-resolution OCT/VLE images. A recent investigation with AI technologies developed an interpretation of the OCT/VLE images. First, Qi et al. demonstrated that automatic interpretation of the OCT images for diagnosing dysplastic lesions in BE patients had a sensitivity and specificity of 82% and 74%, respectively [40,102]. Next, Leggett et al. revealed that the diagnostic accuracy for Barrett’s dysplasia by the CAD system of the VLE imaging (CAD-VLE) was better than those by manual diagnosis using the criteria of a single-center study with 50 VLE datasets for 50 EMRs, whose specimens were imaged with VLE and classified histologically into a neoplastic category (HGD/IMC) and a non-neoplastic category (LGD/ NDBE) [103]. In detail, the diagnostic accuracy of the CAD-VLE had a sensitivity of 86%, specificity of 88%, and accuracy of 87%. Further studies will be needed to develop AI systems to interpret VLE imaging.

#### 2.4.2. CAD for FME

The AI system assisting in the diagnosis process of molecular imaging for dysplastic BE has been innovated by the development of an accurate measurement of fluorescence intensity. Jiang et al. developed an image processing algorithm, the Chan-Vese algorithm, for a wide-field FME to provide guidance for endoscopic biopsy or treatment in BE [40]. Although the low-contrast and non-uniform signals of the tracers with a patchy distribution in large Barrett’s mucosa may cause an unsatisfactory evaluation by conventional segmentation algorithms, the highlighted region in a noisy low-contrast background was analyzed by the Chan-Vese algorithm to achieve a high T/B ratio in the ROIs. With 50 datasets recorded in Barrett’s surveillance, the algorithm made an automated diagnosis of HGD/EAC with a sensitivity and specificity of 92% and 75%, respectively.

Joshi et al. identified HGD/EAC lesions by assessing the T/B quantification, which might highlight targeted regions with high fluorescence signals of ASYNYDA above the threshold intensity of HGD/EAC based on the established algorithm, with a sensitivity of 76% and specificity of 94% after postprocessing the fluorescence video frames from a wide-field custom fluorescence image [79].

## 3. Another Issue in Diagnosing Barrett’s Neoplasia

Assessment of the histology and advanced imaging technologies have not been validated for Barrett’s LGD, although LGD may be a point for endoscopic intervention. In a Dutch study, 73% of BE cases with histological diagnosis of LGD were down-staged to NDBE or indeterminate BE after histological review [3]. Further development of advanced imaging with molecular imaging technologies is expected to resolve the issue of endoscopic and histopathological assessment of LGD.

## 4. Perspective

Recent advances in endoscopic technologies might visualize morphological and biological alterations during Barrett’s cancer development. Molecular imaging with multiple biomarkers specific to Barrett’s HGD/EAC may soon be able to detect lesions at an early stage and predict treatment response for precision medicine. Currently, such technologies have not yet become clinically widespread because of the limited view field, expense of equipment, long learning curve, and insufficient evidence. Moreover, the development of fluorescent tracers for molecular imaging is costly and requires laborious steps for reproducible labeling methods, as well as stability and toxicity studies. However, further developments in the combinations of advanced endoscopic imaging technologies with specific biomarkers and the CAD system should enable us to realize accurate and real-time diagnosis of neoplastic BE surveillance in the future.

## 5. Conclusions

The current surveillance strategy with endoscopy and random biopsies for BE patients has some issues concerning sensitivity, specificity, and accuracy for diagnosing dysplasia/EAC due to sampling errors and inter/intra-observer variation. Clinically available IEE imaging technologies are sensitive enough to detect small foci of dysplasia, which might be inconspicuous for HRWLE, but issues concerning low specificity with relatively large variance in interpretation of the findings remain. Advanced imaging techniques with specific biomarkers may identify morphological abnormalities and biological disorders, enabling early intervention for Barrett’s neoplasia to target malignant foci, reducing sampling error, improving the efficacy of surveillance with risk stratification, and providing treatment efficacy for precision medicine. Further research is needed to develop new endoscopic modalities with high sensitivity and specificity for dysplasia detection in cost-effective population-wide BE surveillance.

## Abbreviations

BE	Barrett esophagus
EAC	esophageal adenocarcinoma
HRWLE	high-resolution white-light endoscopy
SP	the Seattle protocol
CLE	confocal laser endomicroscopy
AFI	autofluorescence imaging
OCT	optical coherence tomography
AUC	an area under the receiving operator characteristics curve
FISH	fluorescence in situ hybridization
CAD	computer-assisted diagnosis
VLE	volumetric laser endomicroscopy
VLEL	a VLE laser marking system
ROI	regions of interest
AI	artificial intelligence
IEE	image-enhanced endoscopy
NBI	narrow band imaging
MNBI	high-definition magnification NBI
ETMI	endoscopic trimodal imaging
CLE	confocal laser endomicroscopy
pCLE	the probe-based CLE
eCLE	the endoscopy-integrated CLE
NIR	near-infrared range
FME	fluorescence molecular endoscopy
NPs	nanoparticles
WGA	wheat germ agglutinin
T/B ratio	tumor-to-background ratio
ASYNYDA	sequences ASY*-fluorescein isothiocyanate
Hsp-TPP	membrane-bound Hsp70-specific contrast agent Tumor-Penetrating peptide
IHC	the immunohistochemical study
NDBE	non-dysplastic Barrett esophagus
LGD	low-grade dysplasia
HGD	high-grade dysplasia
EGFR	epidermal growth factor receptor
HER2	human epidermal growth factor receptor
NIR-FME	wide-field near-infrared FME
VEGFA	vascular endothelial growth factor
CXCR4	chemokine receptor 4
PpIX	protoporphyrin IX
2NBDG	2-[N-(7-nitrobenz-2-oxa-1,3-diaxol-4- yl)amino]-2-deoxyglucose
GLUT	glucose transporters
